# Acceleration of phenological advance and warming with latitude over the past century

**DOI:** 10.1038/s41598-018-22258-0

**Published:** 2018-03-02

**Authors:** Eric Post, Byron A. Steinman, Michael E. Mann

**Affiliations:** 1 Department of Wildlife, Fish, & Conservation Biology, University of California, Davis, One Shields Avenue, Davis, CA 95616 USA; 2 Department of Earth and Environmental Sciences and Large Lakes Observatory, University of Minnesota Duluth 2205 E. 5th Street, Research Laboratory Building, Duluth, MN 55812 USA; 3 Department of Meteorology and Atmospheric Science, and Earth & Environmental Systems Institute, Pennsylvania State University, University Park, PA 16802 USA

## Abstract

In the Northern Hemisphere, springtime events are frequently reported as advancing more rapidly at higher latitudes, presumably due to an acceleration of warming with latitude. However, this assumption has not been investigated in an analytical framework that simultaneously examines acceleration of warming with latitude while accounting for variation in phenological time series characteristics that might also co-vary with latitude. We analyzed 743 phenological trend estimates spanning 86 years and 42.6 degrees of latitude in the Northern Hemisphere, as well as rates of Northern Hemisphere warming over the same period and latitudinal range. We detected significant patterns of co-variation in phenological time series characteristics that may confound estimates of the magnitude of variation in trends with latitude. Notably, shorter and more recent time series tended to produce the strongest phenological trends, and these also tended to be from higher latitude studies. However, accounting for such variation only slightly modified the relationship between rates of phenological advance and latitude, which was highly significant. Furthermore, warming has increased non-linearly with latitude over the past several decades, most strongly since 1998 and northward of 59°N latitude. The acceleration of warming with latitude has likely contributed to an acceleration of phenological advance along the same gradient.

## Introduction

Advancing spring phenology constitutes one of the most well studied and oft-cited examples of ecological response to recent climatic warming^1^. Examples of earlier onset of spring events span a broad array of life history traits and taxonomic groups, including plants, invertebrates, amphibians, birds, and mammals across most of Earth’s biomes. For instance, earlier onset of spring events such as green-up and flowering in plants, migratory arrival in birds, calling and spawning in amphibians, and emergence in insects have been reported in the primary literature and several major syntheses of ecological responses to observed warming over the past two decades^2–7^, including the most recent IPCC Fifth Assessment Report^8^. In addition to documenting generally, though not universally, earlier onset of spring events in response to warming, one of the major insights of such work has been more rapid advancement of spring events at higher than at lower latitudes^5,9^.

Such a latitudinal gradient in phenological trends presumably relates primarily to the convergence of two related phenomena. First, species with latitudinally broad geographical distributions tend to exhibit latitudinal gradients in the timing of expression of life history traits, with timing of events generally occurring later as latitude increases. Numerous examples of this pattern exist^5^, and include, among many others, latitudinal gradients in arrival timing and nesting in birds^10–12^; nesting in sea turtles^13^; emergence and flight phenology in insects^14^; and extensively documented latitudinal variation in the timing of initiation of growth, flowering, and fruiting in plants^15–21^. This pattern ostensibly relates to the inter-related constraints of latitudinal variation in local temperature and seasonal day length on phenology^22^. Second, the magnitude of recent land surface warming has been generally greater at higher than at lower latitudes^23^. Hence, the alleviation of temperature constraints on the annual timing of expression of life history traits due to climate change has apparently contributed to or amplified latitudinal variation in phenology^24^.

Taken together, these observations suggest that continued anthropogenic warming and resulting record temperatures^25^ might elicit even greater phenological advances at higher than at lower latitudes, unless resulting conditions become constraining^26^. But this expectation has not yet been informed by an integrative, quantitative assessment of variation in rates of phenological advance and warming with latitude. To date, support for the notion that rates of phenological advance have been greater at higher than at lower latitudes, and that this difference is due to more pronounced warming at higher latitudes, has been largely piecemeal. Previous syntheses and meta-analyses have relied upon: (1) binned analyses, in which phenological trends are compared between low- and high-latitude studies or among latitudinal zones^6,7,17^; (2) simple correlations between rates of phenological advance and latitude that have not accounted for potentially confounding variation due to taxonomic or trait-based differences^5^; or (3) insights gleaned from qualitative comparisons among rates reported in the literature^8,9^.

Here we assess latitudinal variation in rates of phenological advance in an analytical framework that accounts for continuous spatial variation and potentially confounding covariates related to characteristics of the focal phenology time series. This approach departs from previous assessments that have, for instance, examined latitudinal variation in phenological trends while combining trend data derived from an array of different phenological events and taxonomic groups studied over widely disparate periods throughout the 20^th^ Century and into the 21^st^ Century. We furthermore complement our analysis with one of spatio-temporal variation in patterns of Northern Hemisphere land-surface warming over the past century to investigate the extent to which the rate of warming over this period has varied with latitude.

## Methods

### Variation in rates of phenological change with latitude

We compiled rates of phenological change from primary sources reviewed in three previous major syntheses and meta-analyses that included assessments of latitudinal gradients^5,6,9^. In addition to these, we gathered rates of phenological change from additional primary sources, and in this case selected rates based on time series of observational data spanning at least ten years. This resulted in a pool of 743 estimates of rates of phenological change, including both significant and non-significant trends. These trends span a latitudinal gradient from 31.9°N to 74.5°N, and encompass studies beginning between 1928 and 2002 and ending between 1978 and 2013. Hence, the geographic extent of the meta-data used here includes 42.6 degrees of latitude, and the temporal extent, from earliest to latest observations upon which trend estimates are based, includes 86 years. Taxa include amphibians, birds, invertebrates, mammals, and plants. A complete list of the published rates of phenological change and their sources, as well as descriptive meta-data including taxon, latitude, and time series dates and lengths included in this analysis is provided in the online supplementary material.

To analyze variation in rates of phenological change (i.e., phenological trends indicating advance or delay), in association with latitude, we first employed simple linear regression between published phenological trends expressed as days/decade and latitude of the individual study location as reported in the primary source. We then conducted additional analyses to account for potential contributions of other factors to the relationship between rate of phenological change and latitude. These included time series characteristics (the first year of observation and length of the time series upon which rate estimates were based), taxon, and phenological trait. To accomplish this, we applied a generalized additive model (GAM)^27^. GAMs allow for simultaneous consideration of dependent variable responses to continuous numerical predictors and categorical predictors in the same model, and are therefore occasionally referred to as “categorical regression models”^27^. Our GAM included latitude and time series characteristics as continuous numerical predictors, and taxon and phenological trait as nominal categorical predictors. The meta-data pool included both significant and non-significant phenological trend estimates. Significance (two-tailed *p* ≤ 0.05) was determined on the basis of *p-*values accompanying trend estimates based on simple linear correlations or linear regression coefficients in the original sources from which the trends were obtained. Previous meta-analyses have focused on analyzing variation in significant trends only^6^ because, technically, non-significant trends do not differ from zero. We analyzed pooled significant and non-significant trend estimates and accounted for significance by including in our GAMs a nominal categorical predictor, “significance”. However, for comparison, we also report results of analyses focusing on significant phenological trends only.

### Variation in rates of land surface warming with latitude

Northern Hemisphere land surface temperature data were obtained from the University of Delaware (UDel) surface temperature record v301, for the period 1928–2010, and from latitudes 32.25°N through 74.25°N, to most closely overlap the temporal and spatial extent of the phenology meta-dataset described above. We used monthly mean temperatures at a spatial resolution of 1° latitude for the boreal spring months April through June to derive annual estimates of boreal spring temperature anomalies at each one-degree latitudinal band. Spring temperature anomalies were calculated as departures from the 1951–1980 mean for the April through June period at each latitudinal band.

To investigate whether the rate of spring warming in the Northern Hemisphere has increased with latitude and through time for the spatio-temporal domain considered here we employed a multi-stage approach. First, we analyzed trends in temperature anomalies for the boreal spring at 1° latitudinal increments over the entire period, from 1928 through 2010, and then for successively more recent periods in one-decade intervals (i.e., 1938–2010, 1948–2010, 1958–2010, 1968–2010, 1978–2010, 1988–2010, and 1998–2010). We justify this approach on the basis of the fact that, as described above, the phenological trend estimates used here derive from studies conducted over periods of various lengths and with various start years throughout the 20^th^ Century and early 21^st^ Century (see also metadata supplement). Hence, this approach was intended to investigate and quantify accelerating trends in the rate of warming with latitude, and replicates, in the temporal domain, previous analyses demonstrating increasing rates of global surface warming in more recent multi-decadal periods since the mid-1800s^8,23^. We achieved this by deriving estimates of the slope of the linear regression between spring temperature anomalies and “year” for each period. Next, we analyzed variation in these trend estimates with latitude, accounting for potentially non-linear associations between rate of land surface warming and latitude by comparing linear and non-linear (quadratic) regression models. Our determination of the best-fit model is based on a balance among parsimony, *F*-values, and visual inspection of the fit of the model to the data. If the coefficient of determination increased by less than ten percent from the linear to the quadratic model, in the interest of parsimony we did not regard this as indicative of a better model. If non-linearity in this association was indicated, we subsequently employed piecewise non-linear (threshold) modeling to identify the threshold latitude below and above which the rate of warming differed within any given period, including the full set of temperature anomaly data (1928–2010) and each more recent decadal period up to 1998–2010. Because 1998 included a strong El Niño event, and was notable for its exceptional global land-surface temperature anomaly, we repeated our analyses using 1997–2010 and 1999–2010 as the most recent period. Results are presented using all three intervals for the most recent period. To determine whether variation in rates of warming with latitude differed significantly among more recent decadal periods, we performed an ANOVA with trends in spring warming as the dependent variable, the start year of the period of consideration as a fixed factor, and latitude as a covariate.

Finally, as noted above, we chose the (UDel) gridded temperature data product for our analysis because it is one of only two temperature datasets that has sufficiently high spatial resolution for our analysis; the other is the Climatic Research Unit (CRU) TS4.01 Mean Temperature dataset^28^. To assess potential differences between the two data products, we regressed the latitudinal mean UDel values onto CRU values for the periods 1928–2010, 1938–2010, 1948–2010, 1958–2010, 1968–2010, 1978–2010, 1988–2010, and 1998–2010. Slope estimates (i.e., beta values) of these regressions displayed no systematic departure from parity with latitude for any of the periods (Supplemental Figure S2). Nonetheless, we note that beta values for the periods 1978–2010, 1988–2010, and 1998–2010 are on average slightly greater than one. However, the departure from parity for these periods, as noted, displays no trend with latitude, and we therefore conclude that applying the CRU dataset to our analyses likely would not alter our conclusions relative to latitudinal gradients.

### Data availability

All data are available on request, and will be archived online for public access prior to publication.

## Results and Discussion

Of the total number of phenological trend estimates included in our analyses, 449 were reported as significant and 294 as non-significant at the *α* = 0.05 level in the primary literature from which they were derived. Hence, greater than one-third (39.6%) of published phenological trends considered in this sample constitute stasis, or a lack of significant directional change. Significant phenological trends varied from a maximum delay of 7 days/decade to a maximum advance of −36.3 days/decade, while non-significant trends varied from a maximum delay of 26.3 days/decade to a maximum advance of −51.3 days/decade. Among significant phenological trends, 415 (92.4%) were negative, indicating an overwhelming tendency toward phenological advance. The mean (±1SE) rate of advance among significant phenological trends was −6.40 (±0.34) days/decade, while the mean rate of delay among significant phenological trends was 3.12 (±0.36) days/decade, indicating that on average phenological advances, where these have occurred, have been more than twice as great as phenological delays.

**Figure 1:**
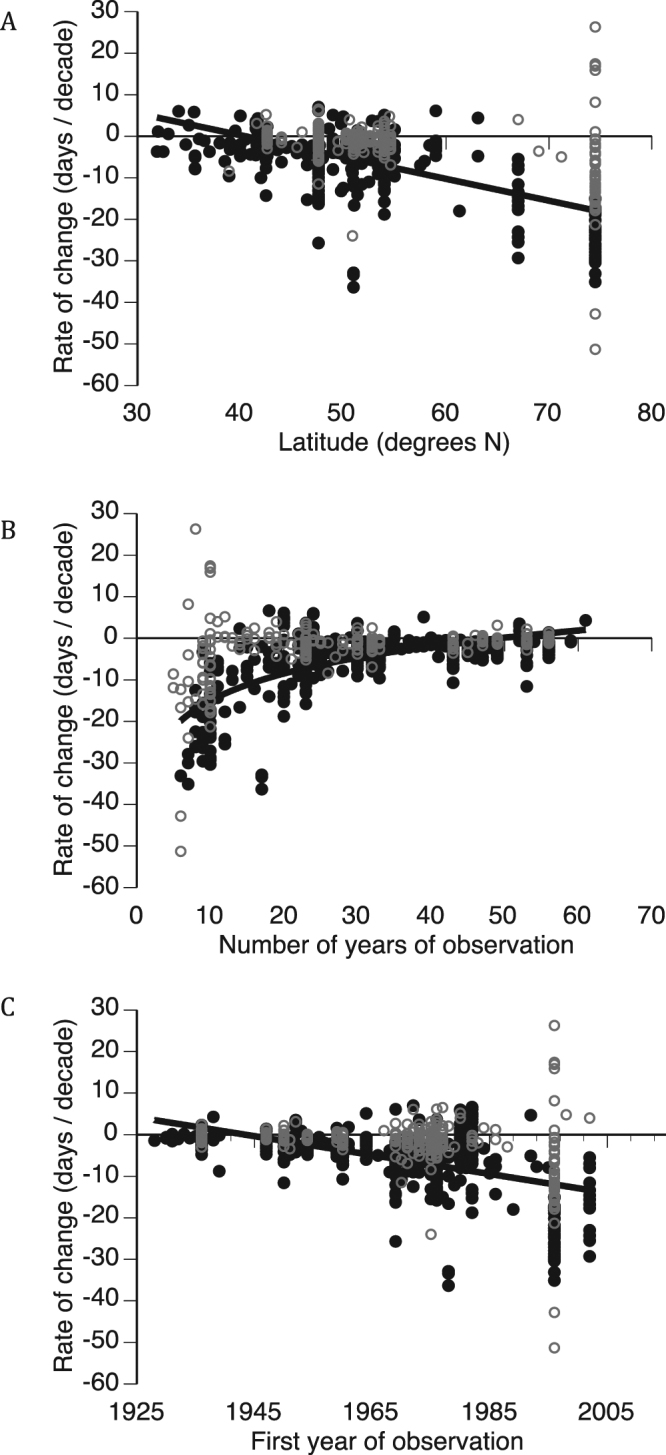
Relationships between phenological trends (positive = later, or delayed, occurrence; negative = earlier, or advanced, occurrence) and degrees north latitude (**A**), length of the time series upon which trend estimates are based (**B**), and the first year of observation in the time series upon which trend estimates are based (**C**). Non-significant (*P* > 0.05) trend estimates are shown in gray; black dots indicate significant trend estimates in all panels. Trend lines in each panel apply to scatter plots of significant trend estimates only. In panel A, the slope of the relationship is −0.50 ± 0.04 for pooled significant and non-significant phenological trends, and −0.53 ± 0.03 for significant phenological trends only.

Pooled rate estimates, including both significant and non-significant rates, displayed substantial heteroscedasticity in association with latitude, time series length, and the first year of observation in the time series from which they were derived (Fig. 1). In other words, variability among estimates of phenological trends increased with study site latitude, and was greater among shorter and more recent studies. The simple GAM of pooled significant and non-significant phenological trends with latitude as the sole numerical predictor revealed a significant increase in rate of phenological advance with latitude (Fig. 1A; standardized beta = −0.50 ± 0.04, *P* < 0.001) even after accounting for categorical significance (standardized beta = −0.40 ± 0.02, *P* < 0.001). The slope of the relationship with latitude suggests that the decadal rate of advance in springtime events represented in this meta-data pool increases by approximately 0.4 to 0.5 days/decade with each one-degree increase in north latitude. For comparison, an analysis of statistically significant phenological trends only revealed a slightly stronger association with latitude (standardized beta = −0.53 ± 0.03, P < 0.001).

The rate of increase in phenological advancement with latitude revealed by the simple GAM of pooled significant and non-significant trend estimates is greater than the only other such reported relationship that we are aware of^5^. However, these relationships are not strictly comparable. Although our meta-data pool includes the phenological trend estimates used in Parmesan (2007), our analysis also included slope estimates that were published subsequent to the analysis by Parmesan (2007), including additional estimates from high latitude studies.

The more complex GAMs of pooled significant and non-significant phenological trends that included time series characteristics as co-variates furthermore revealed significant associations between rate of phenological advance and time series length (standardized beta = 0.36 ± 0.05, *P* < 0.001) and first year of observation (standardized beta = −0.26 ± 0.03, *P* < 0.001). Among significant phenological trends only, trend estimates were strongly and non-linearly related to time series length (Fig. 1B), and linearly related to the first year of observation (Fig. 1C). The former relationship (log-linear regression *R*^2^ = 0.45, *P* < 0.001) indicates that short-term observational studies tend to produce strongly negative estimates of rates of phenological change, i.e., phenological advance, while longer studies tend to produce more modest, and occasionally positive, trend estimates (Fig. 1B). The latter relationship (linear regression *R*^2^ = 0.29, *P* < 0.001) indicates that more recent studies have also tended to produce strongly negative estimates of rates of phenological change, while earlier studies have tended to produce weaker trend estimates (Fig. 1C). Notably, the rate of global surface warming has increased recently, which might contribute to the greater rates of phenological advance in more recently initiated studies. For example, the rate of increase in global mean temperature (°C/decade) from 1981–2005 was nearly four times as great as the rate of warming from 1856–2005^23^.

**Figure 2:**
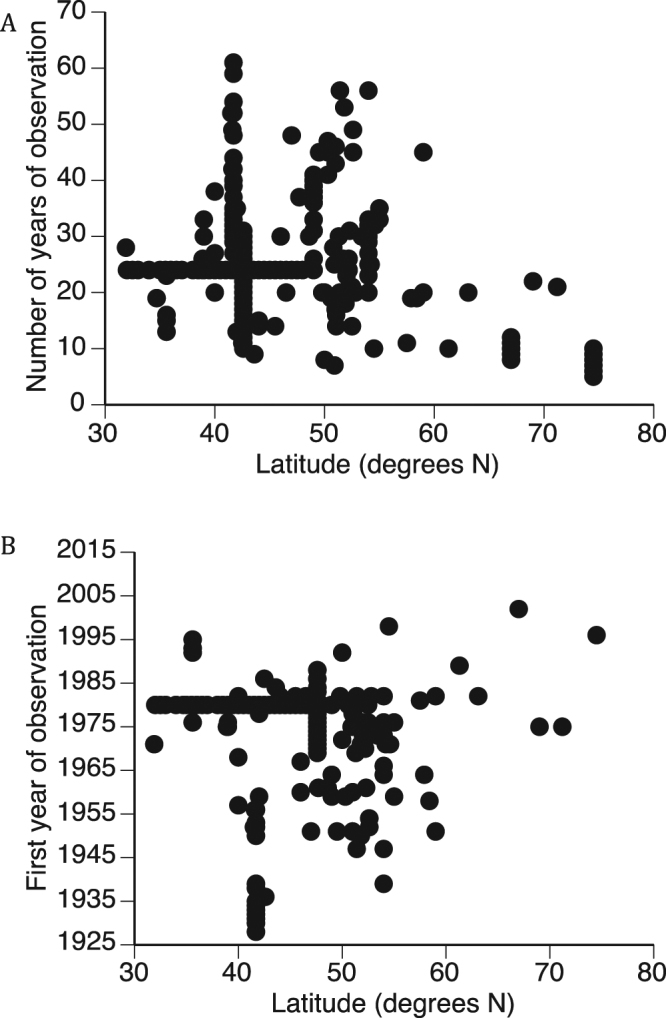
Variation with latitude in time series length (**A**) and the first year of observation (**B**) for studies upon which phenological trend estimates used in this analysis were based. Both panels include studies reporting statistically significant and non-significant phenological trends. Hence, high latitude studies of phenology have been of significantly shorter duration and more recent than lower latitude studies.

Perhaps unsurprisingly, time series length and the first year of observation in the studies considered here are negatively related (*r* = −0.65, *P* < 0.001). More importantly in the context of the central question motivating our analyses, both time series length and the first year of observation upon which phenological trend estimates were based were significantly related to latitude. Far northern observations of phenology have been shorter than those at lower latitudes (*r* = −0.38, *P* < 0.001) because they have been initiated more recently (*r* = 0.59, *P* < 0.001) (Fig. 2). Hence, an important consideration when interpreting latitudinal gradients in phenological trends is whether these relate to statistical artifacts associated with sample size, or whether they relate to greater rates of warming at higher latitudes.

Because of the correlation between the first year of observation and time series length in the phenological studies considered here, these two variables cannot be included together in a multivariate model of phenological trends. For this reason, the more complex GAMs reported above were conducted individually, each with either start year or time series length as a predictor, and both with the additional predictors: latitude, taxon, significance category, and the phenological trait upon which the trend estimate was based. In the GAM with start year as a predictor, phenological trend remained significantly negatively related to latitude (standardized beta = −0.38 ± 0.04, *P* < 0.001) after accounting for significant associations with first year of observation (standardized beta = −0.26 ± 0.03, *P* < 0.001) and with significance category, taxon, and trait (all *P* < 0.001; full model *R*^2^ = 0.50, *P* < 0.001). The GAM with time series length as a predictor revealed a stronger, significantly negative relationship between phenological trend and latitude (standardized beta = −0.40 ± 0.05, *P* < 0.001), and a significantly positive association between phenological trend and time series length (standardized beta = 0.36 ± 0.05, *P* < 0.001), with significance category, taxon, and trait remaining significant (all *P* < 0.001). However, this second GAM explained 9% more of the variation in phenological trend estimates (full model *R*^2^ = 0.59, *P* < 0.001). Hence, even after accounting for variation due to time series metrics and taxonomic- and trait-based differences among studies, which no previous study has to our knowledge attempted, the increase in rate of phenological advance with latitude remained strongly significant.

**Figure 3:**
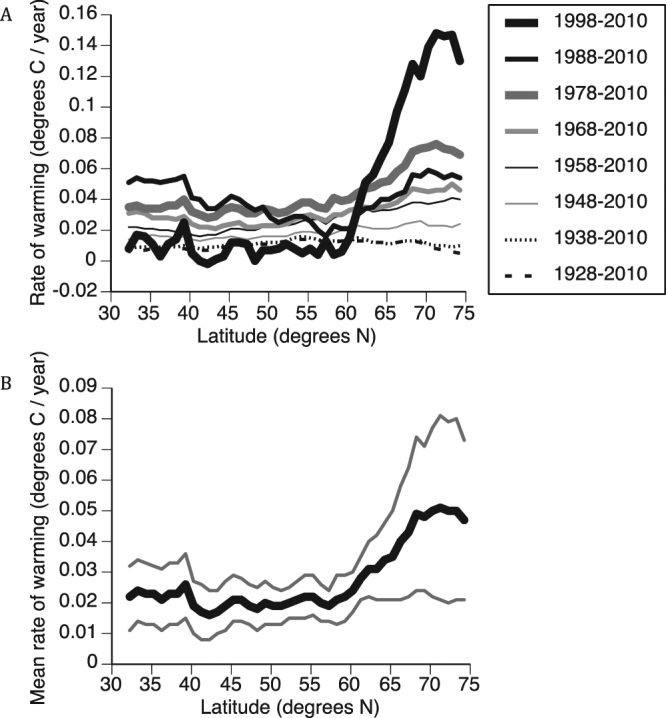
(**A**) Variation in the relationship between rate of Northern Hemisphere springtime land surface warming and latitude among successively more recent decadal periods beginning in 1928, coinciding with the periods encompassed by the studies upon which estimates of phenological trends used in this analysis have been based. (**B**) The mean rate (heavy line) and upper- and lower 95% confidence limits (thin lines) of Northern Hemisphere springtime land surface warming calculated across all periods in panel (**A**).

The rate of boreal spring land surface warming varied significantly with latitude over the period 1928–2010 and all more recent decadal periods (Supplemental Table S1). Visual inspection of the data, together with comparisons among linear and non-linear quadratic regression models, indicated that the rate of warming increased linearly with latitude only during the periods 1948–2010 and 1958–2010 (Table S1). In all periods from 1968 onward, the rate of warming was strongly non-linearly related to latitude (Table S1), increasing northward of approximately 57°N (Fig. 3A). The most recent period of warming, 1998–2010, also displayed the strongest departure from linearity, with the rate of warming increasing dramatically with latitude northward of approximately 59°N (Fig. 3A). Indeed, a piecewise linear approximation of a threshold non-linear model centered on 59°N latitude revealed a non-significant relationship between rate of warming and latitude below this threshold (standardized beta = −0.26, *P* = 0.20) but a highly significant positive relationship at and above it (standardized beta = 0.96, *P* < 0.001). Delimiting this period as 1997–2010 or 1999–2010 also revealed strong departures from linearity in the relationship between rate of warming and latitude at a threshold of 59°N (Supplemental Figure S1).

Hence, the relationship between rate of land surface warming and latitude in the Northern Hemisphere is highly variable, and characterized by varying degrees of non-linearity, among the successively more recent periods of the past century examined here (Fig. 3A, Table S1). Indeed, our ANOVA indicates a highly significant effect of period in the relationship between rate of warming and latitude (*F*_1,43_ = 152.3, *P* < 0.001). The mean rate of warming calculated across all of these periods suggests, additionally, a generally non-linear increase in rate of warming with latitude (Fig. 3B). In support of this, a non-linear (quadratic) model provided substantially better fit to the relationship between rate of warming and latitude than did a linear model (*R*^2^_linear_ = 0.61, *F*_linear_ = 63.8, *P*_linear_ < 0.001; *R*^2^_quadratic_ = 0.93, *F*_*quadratic*_ = 267.3, *P*_*quadratic*_ < 0.001).

Our analyses have revealed significant increases in rates of phenological advance with latitude both before and after accounting for variation in such rates attributable to taxonomic representation, life history traits, and timing and duration of observational studies from which they derive. These results have also raised a cautionary note that higher latitude studies considered here and elsewhere might produce trend estimates indicating greater rates of phenological advance than those at lower latitudes simply because they have been shorter in duration. However, our analyses also indicate that shorter studies have generally been more recent and likely have revealed greater rates of phenological advance at high latitudes because these studies coincide with the period during which warming has increased most strongly and non-linearly with latitude. Indeed, of the four arctic studies considered here, the two that reported significant rates of phenological advance commenced in 1996^29^ and 2002^30^, largely coincident with the onset of the rapid increase in rates of warming with latitude (Fig. 3A), while the two that reported non-significant rates of advance commenced in 1975. Unless limits to phenological plasticity eventually establish^31^, further acceleration of rates of warming with latitude may pose consequences for long distance migrants that track resource phenology along latitudinal gradients.

## Electronic supplementary material

Supplemental Table S1 and Supplemental Figures S1 and S2

Dataset 1
