# Supplementary material for: Acceleration of phenological advance and warming with latitude over the past century
Source: Sci Rep. 2018 Mar 2;8:3927. doi: 10.1038/s41598-018-22258-0 (PMC5834618; doi:10.1038/s41598-018-22258-0)
Supplement: Supplementary file 1 — Supplemental Table S1 and Supplemental Figures S1 and S2 [file 41598_2018_22258_MOESM1_ESM.docx]

**Supplemental online material**

Acceleration of phenological advance and warming with latitude over the past century

Eric Post, Byron A. Steinman, and Michael E. Mann

**Table S1.** Coefficient of determination (*R*^2^) values and *F*-statistics for linear (L) and non-linear (quadratic; Q) regression models of variation in Northern Hemisphere April – June land surface temperature anomalies with latitude. See Methods for details on the source of temperature data and derivation of anomalies used in this analysis. Significant models are indicated in bold font. For quadratic models, coefficients of determination are reported as adjusted *R*^2^ values to account for multiple independent terms. The best-fit model for each period, determined on the basis of a balance among parsimony, *F*-values, and visual inspection of the fit of the model to the data, is indicated in blue font.

| Period | *R*^2^_L_ | *F*_L_ | *R*^2^_Q_ | *F*_Q_ |
| --- | --- | --- | --- | --- |
| 1928 – 2010 | **0.40** | **26.9** | **0.75** | **62.6** |
| 1938 – 2010 | **0.22** | **11.4** | **0.58** | **30.3** |
| 1948 – 2010 | **0.78** | **147.1** | **0.83** | **101.9** |
| 1958 – 2010 | **0.85** | **225.5** | **0.93** | **284.9** |
| 1968 – 2010 | **0.58** | **55.9** | **0.93** | **282.3** |
| 1978 – 2010 | **0.67** | **83.4** | **0.93** | **274.0** |
| 1988 – 2010 | 0.00 | 0.006 | **0.75** | **64.1** |
| 1998 – 2010 | **0.63** | **69.3** | **0.92** | **242.2** |


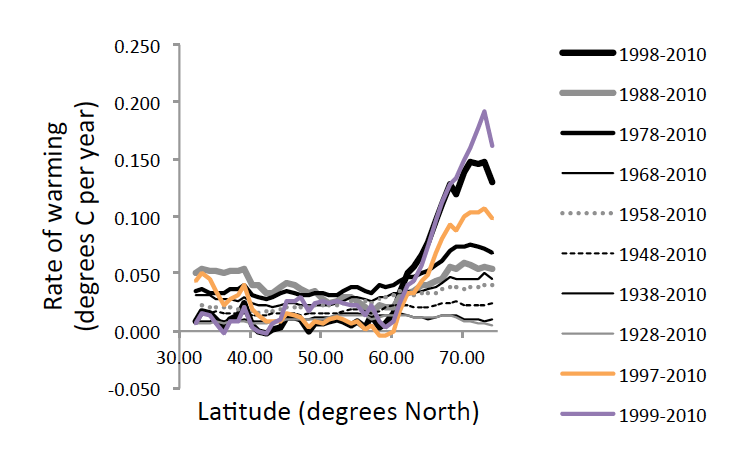
Figure S1. As in Figure 3 of the main text, variation in the relationship between rate of Northern Hemisphere land surface warming and latitude among successively more recent periods beginning in 1928, but with inclusion of rates when the most recent period is defined as 1997 - 2010 (orange line) or 1999 - 2010 (purple line), for comparison to 1998 - 2010 (heaviest black line). Defining the most recent period in either of these two ways does not alter the conclusion of strong non-linearity in the relationship between rate of warming and latitude at a threshold of approximately 59^o^N. For 1997 - 2010, below this latitude the relationship between rate of warming and latitude is strongly negative (standardized beta = -0.85, *P* < 0.001), while from 59^o^N and northward it is strongly positive (standardized beta = 0.96, *P* < 0.001). For 1999 - 2010, below 59^o^N, this relationship is weakly positive (standardized beta = 0.53, *P* = 0.005), while from 59^o^N and northward it is strongly positive (standardized beta = 0.99, *P* < 0.001).

Figure S2. Slope estimates (beta values) from linear regression models of UDel land surface temperature data vs. CRU land surface temperature data at 1^o^ north latitude increments for a) 1928 - 2010, b) 1938 - 2010, c) 1948 - 2010, d) 1958 - 2010, e) 1968 - 2010, f) 1978 - 2010, g) 1988 - 2010, and h) 1998 - 2010. Blue lines represent slope estimates based on 1^o^ spatial resolution, and red lines represent slope estimates based on 5^o^ spatial resolution. In each panel, the horizontal line indicates parity (i.e., a regression slope of 1).
